# Contribution on Yellow Fever

**Published:** 1874-04

**Authors:** 


Contributions to the Natural History of Specific Yellow Fever, Composition and Character of the Urine in Yellow Fever. By Joseph Jones, M.D., Professor, etc. Pamphlet, pp. 52.

We learn that Professor Jones has in contemplation the publication, at an early day, of the results of his multiplied labors and reseaches. It is proposed to issue the work in three volumes, the first of which will be ready for press about the 1st of January next, and the remaining volumes will soon follow. Subscriptions for the work will be received, in advance, at *five dollars* per volume, which will barely cover the actual cost of publication. Prof. Jones has been for years a modest, retiring, but most earnest and indefatigable worker in the field of scientific investigation, and we trust he will be liberally encouraged by the profession in his proposed plan for laying the fruits of his self-sacrificing labor before the scientific world.

---

Headache After Drunkenness.—Byron recommends "sermons and soda water." The *Revue de Therapeutique* says: Take of solution of acetate of ammonia, tincture of bitter orange peel, syrup of bitter orange peel, each 20 parts; water 500 parts. To be given in repeated tablespoonful doses.

Dr. Burdon-Sanderson, in making some experiments on the electric phenomena which accompany the contraction of the leaves of a sensitive plant, found the currents produced are subject in all respects to the same laws as those of muscles and nerves.

